# When a Toolkit Is Not Enough: A Review on What Is Needed to Promote the Use and Uptake of Immunization-Related Resources

**DOI:** 10.9745/GHSP-D-23-00343

**Published:** 2024-02-28

**Authors:** Samantha Jaffe, Ankita Meghani, Jessica C. Shearer, Ami Karlage, Megan B. Ivankovich, Lisa R. Hirschhorn, Katherine E.A. Semrau, Erin McCarville

**Affiliations:** a Population Reference Bureau, Washington, DC, USA.; b PATH, Seattle, WA, USA.; c Ariadne Labs at Brigham and Women’s Hospital and the Harvard T.H. Chan School of Public Health, Boston, MA, USA.; d Northwestern University Feinberg School of Medicine, Chicago, IL, USA.

## Abstract

**Introduction:**

Evidence-based resources, including toolkits, guidance, and capacity-building materials, are used by routine immunization programs to achieve critical global immunization targets. These resources can help spread information, change or improve behaviors, or build capacity based on the latest evidence and experience. Yet, practitioners have indicated that implementation of these resources can be challenging, limiting their uptake and use. It is important to identify factors that support the uptake and use of immunization-related resources to improve resource implementation and, thus, adherence to evidence-based practices.

**Methods:**

A targeted narrative review and synthesis and key informant interviews were conducted to identify practice-based learning, including the characteristics and factors that promote uptake and use of immunization-related resources in low- and middle-income countries and practical strategies to evaluate existing resources and promote resource use.

**Results:**

Fifteen characteristics or factors to consider when designing, choosing, or implementing a resource were identified through the narrative review and interviews. Characteristics of the resource associated with improved uptake and use include ease of use, value-added, effectiveness, and adaptability. Factors that may support resource implementation include training, buy-in, messaging and communication, human resources, funding, infrastructure, team culture, leadership support, data systems, political commitment, and partnerships.

**Conclusion:**

Toolkits and guidance play an important role in supporting the goals of routine immunization programs, but the development and dissemination of a resource are not sufficient to ensure its implementation. The findings reflect early work to identify the characteristics and factors needed to promote the uptake and use of immunization-related resources and can be considered a starting point for efforts to improve resource use and design resources to support implementation.

## Abstract (precis)

While resources like toolkits and guidance play an important role in promoting adherence to evidence-based practice in the administration of routine immunization programs, simply creating these resources is not enough. More work is needed to understand how to promote uptake and use of these resources by routine immunization programs.

## Teaser

**Key Findings:**

- The uptake and use of immunization-related resources are affected by many characteristics beyond evidence-based content, including ease of use and design.
- Adaptability to local context is an important factor for resource use, so ensuring that the resource can be adapted based on input from users on their needs and perspectives, relevance, and alignment with local policies and practices can support resource use.
- When implementing a resource, implementers should invest in sufficient training and capacity-building (e.g., human resources, funding, and infrastructure) for end users to be prepared to use the resource and ensure there is sufficient user buy-in, leadership support, and political commitment.

**Key Implications:**

- Global health practitioners who use toolkits, guidance, and other capacity-building materials to support programmatic goals should consider whether the resource or the context in which it is being implemented supports its uptake and use.
- Practitioners can use the characteristics and factors identified in this article to develop context-specific strategies to ensure optimal uptake and use of resources and to evaluate existing resources for use.

## INTRODUCTION

National routine immunization programs provide lifesaving vaccinations for a country’s maternal, infant, child, and adolescent populations. With more than 20 routine vaccines available that are effective in reducing the risk of morbidity and mortality from acute illnesses, access to these vaccines is critical to ensuring health and well-being, especially among vulnerable and high-risk populations.^1^ Routine immunization programs play an essential role in increasing vaccine uptake, introducing new vaccines, and eliminating diseases targeted for eradication.^2^ However, there are many challenges to implementing routine immunization programs in low- and middle-income countries (LMICs), such as weak national regulatory authorities and systems, constrained resources, lack of education on immunization, and insufficient training of health workers.^3^ Therefore, more guidance and support are needed to ensure immunization programs are implemented effectively and equitably to meet their objectives. To meet this need, global immunization stakeholders have developed evidence-based resources—including toolkits, guidance, and capacity-building materials—that can be used by routine immunization programs to achieve immunization targets.

Toolkits, guidance, and capacity-building materials are designed to spread information, change or improve behaviors, or build capacity based on the latest evidence and experience.^4^ For the purposes of this review, immunization-related toolkits, guidance, and capacity-building materials will cumulatively be referred to as resources. These resources are intended to help end users (e.g., program managers, policymakers, and relevant stakeholders—including civil society, nongovernmental, and faith-based organizations—at subnational and national levels) develop, design, and implement routine immunization programs and sustain high and equitable coverage. Although these resources are intended to support adherence to best practices, practitioners have indicated that the resources themselves can be challenging to implement—requiring staffing, finances, infrastructure, and capacity-building. Additionally, for a resource to be implemented successfully, critical elements at the national, subnational, local, and facility levels must be in place to support their implementation. However, resources are often not designed with information or strategies for the end users to support their facilitation, adoption, or implementation in practice.^4^ A systematic review by Yamada et al. found that, in addition to a toolkit, supplemental efforts and knowledge are required to ensure that the end user can understand and use the toolkit to its fullest capacity.^5^ Thus, developing immunization-related resources is not enough to ensure adoption of evidence-based practice. While resources offer critical tools and information for practitioners, they may be suboptimally implemented if critical implementation challenges are not identified or addressed.

The challenges associated with implementing an evidence-based resource in practice have been referred to in the implementation science literature as the know-do gap.^6^ Related challenges associated with knowledge translation—or putting knowledge into action—and impact evaluation are also important.^7^ Examples of the know-do gap have been documented across the public health landscape, including insufficient implementation of kangaroo mother care,^8^ unsuccessful implementation at scale of interventions to prevent mother-to-child transmission of HIV,^9^ and poor implementation of malaria counseling and treatment for children.^10^ Although challenges associated with resource implementation are known to practitioners, there remains a dearth of information regarding factors and strategies to ensure optimal uptake and use of immunization-related resources. An analysis of the factors that support and limit the implementation of these resources is critical to address this gap.

There remains a dearth of information regarding factors and strategies to ensure optimal uptake and use of immunization-related resources.

Recognizing the know-do gap in immunization-related resource implementation, the MOMENTUM Routine Immunization Transformation and Equity^11^ project partnered with the Measurement, Adaptive Learning, and Knowledge Management Lab (MAKLab),^12^ as part of the MOMENTUM Knowledge Accelerator project, to identify common barriers and best practices when implementing immunization and related resources. Although practice-based insights are sometimes shared in reviews or reports, this learning has not been consolidated into comprehensive guidance for resource implementation. This article describes the output of a collaborative activity between these MOMENTUM projects^13^ to conduct a targeted narrative review and synthesis and key informant interviews to identify practice-based learning. The review focused on identifying characteristics and factors that promote the uptake and use of immunization-related resources as well as practical strategies that resource users can employ to evaluate existing resources and promote resource use. This article describes the process that the MOMENTUM projects followed to extract relevant findings for use in practice and the key learnings generated through this process.

## METHODS

The activity team consisted of 5 individuals from MAKLab, who led information collection and analysis, and 2 individuals from the MOMENTUM Routine Immunization Transformation and Equity Project, who provided expert input.

There are multiple frameworks within implementation science that define implementation, uptake, and use. For the purposes of this work, implementation was defined as the process of putting an intervention or resource into use.^14^ The components of implementation—uptake and use—describe the adoption and sustained use and acceptance of a resource, respectively.^15^

The activity team conducted a targeted narrative review to identify relevant articles—including published and gray literature (e.g., reports)—on immunization-related resource implementation. Articles were identified through an online search and organized in a Microsoft Excel spreadsheet. For the purposes of this activity, we defined a narrative review as a semisystematic process to search for related literature and extract and synthesize relevant information in a narrative format. The search process evolved as information was collected and was not dictated at the outset by a research protocol. We recorded the process of literature review and information extraction to provide a description of our methods. Although not as comprehensive as a scoping or systematic review, this process allowed for rapid information collection that could inform planned work.

The primary search terms used for the narrative review included immunization, implementation, resources, and LMICs. Variations of each of these primary search terms were also used to ensure completeness of the search. For example, the terms acceptability, use, and feasibility were used interchangeably for the term implementation. Google Scholar was the primary search engine.

The initial search focused on articles on the implementation of specific immunization-related resources, including the Urban Immunization Toolkit^16^ and Reaching Every District guidance.^17^ This search only generated 5 relevant articles; inclusion criteria were subsequently expanded to include articles that evaluated or described barriers, facilitators, and contextual factors affecting uptake or use of resources in LMICs. We included articles for review if (1) they were available in English, (2) the resource of interest met the predetermined definition of a resource, (3) the resource was developed for or used in an LMIC, and (4) the resource was developed for a program or intervention that was immunization related or similar in nature and scope to immunization services. We excluded articles if they did not meet all inclusion criteria.

Two members of the activity team evaluated articles for eligibility using the inclusion criteria described, and articles deemed eligible by both team members were included. Next, the team abstracted information from the articles using an Excel spreadsheet with the following fields: article title, article date, summary of article, target user of resource, country/region, context (urban/rural), resource name, and whether the resource was immunization related (yes/no). Barriers, facilitators, and contextual factors associated with resource uptake and use were summarized for each article in the Excel document. Information was organized using the Consolidated Framework for Implementation Research (CFIR) domains of intervention characteristics, outer setting, inner setting, characteristics of the individual, and process.^18^ CFIR was chosen because it provides a comprehensive framework to systematically identify factors that may influence implementation across multiple levels and within various contexts.^18^

In addition to the narrative review, 2 activity team members (SJ and EM) conducted informational interviews with end-user key informants. We invited via email practitioners with relevant experience implementing immunization-related resources in LMICs to participate in an interview to share their experience. Two interviews were conducted via video conference using a semistructured interview guide, and team members took notes in Microsoft Word. Interviewers asked respondents about their experience implementing immunization-related resources, how resources were used, challenges experienced during resource use, and strategies or adaptations that supported uptake and use. An interviewer reviewed written notes immediately following the interview for accuracy.

The activity team analyzed information from the narrative review and interviews separately, and findings were triangulated to generate a shared list of themes. First, 3 team members (SJ, EM, AK) independently reviewed information abstracted from the narrative review to identify a list of themes within each CFIR domain. The CFIR domains were used to ensure each team member identified and considered all factors of the implementation spectrum. Each team member then categorized the identified themes in a way that would provide practitioners with a useful and clear framework to evaluate immunization-related resources and utilize practice-based strategies to promote uptake and use. The 3 team members met to review themes, discuss points of discrepancy, and align terminology, ultimately generating a comprehensive list of themes from the narrative review. After the key informant interviews were completed, the same team members reviewed the interview notes and identified high-level themes using a deductive approach. Team members assigned codes based on the domains of the CFIR framework. Themes were highlighted and annotated within the interview documents. Team members met to review and align codes generated during the coding process.

Next, team members cross-referenced the themes identified in the interviews with those developed from the narrative review to identify points of convergence and divergence and consolidated findings to generate a list of themes across information sources (narrative review and interviews) associated with the uptake and use of immunization-related resources.

To help make findings actionable for implementers, the team then brainstormed and generated a list of strategies that could be used to promote resource uptake for each theme. Strategies were developed as recommendations to the MOMENTUM Routine Immunization Transformation and Equity Project and were intended as examples to aid practitioners in actualizing findings to facilitate practical information use. They were not designed to be exhaustive of all potential implementation strategies.

### Ethical Approval

The Harvard Human Research Protection Program determined that because this activity was completed for quality improvement purposes, it was not human subjects research necessitating institutional review board approval (protocol #IRB23-0822).

## RESULTS

We identified 24 articles; of those, 11 met the inclusion criteria. All articles were in the context of an LMIC setting and were published between 2005 and 2022, and most (8 of 11) focused on immunization-related resources. We invited 6 practitioners to participate in the informational interview, of whom 3 agreed to participate and 2 completed the interview process (33.3% response rate).

We organized the characteristics and factors identified from the narrative review and key informant interviews into 2 tables. Table 1 lists characteristics to consider when designing or choosing a resource, and Table 2 contains factors to consider when implementing a resource.

**TABLE 1.** Characteristics to Consider When Designing or Choosing a Resource and Strategies to Promote Uptake

**Characteristics of the Resource**	**What Strategies Could Improve Uptake and Use?**
**Ease of use**^19^**^–^**^22^ The end users of the resource perceive the resource to be easy to understand and use. The language is appropriate for the intended audience. The resource has an engaging structure that improves the likelihood of use.	Evaluate the resource for ease of use. Criteria include:− The language is simple and easy to read.− The format/structure is easy to follow.− The content is simple/not overly complex.− Tools are available to support use.− The resource is translated or made available in the local language or preferred language of the end user. Pilot test language and format with local staff. Develop additional implementation tools to support use. Use participatory and collaborative strategies to engage users in resource design.
**Value added**^19^^,^^20^ The resource is perceived as an alternative that improves existing ways of working. The resource complements existing interventions and programming.	Evaluate the resource for value added. Criteria include:− The resource is higher quality than alternatives.− The resource helps improve work.− The resource is compatible with work responsibilities.− The resource is not duplicative of other resources. Pilot test the resource with local workflows.
**Effectiveness**^21^**^,^**^22^ The resource was developed or is supported by a trusted source. The resource delivers on its promises (it appropriately addresses what it claims to address). The resource presents evidence of efficacy of the content or recommendations. The resource makes recommendations based on evidence.	Evaluate the resource for effectiveness. Criteria include:− The content is supported by evidence.− The resource was developed by a credible source.− There is evidence of resource efficacy.
**Adaptable with local input**^19^**^–^**^21^**^,^**^23^ The resource acknowledges that adaptations may be needed to enable implementation in different contexts. The resource allows for some modification. The resource provides guidance on how adaptations should be made.	Evaluate whether the resource allows for adaptation. Evaluate whether the resource provides guidance on how modifications can be made. Seek input from local users to adapt the resource. Identify local adaptations that may be helpful. Adapt the resource to the local context if appropriate.

**TABLE 2.** Factors to Consider When Implementing A Resource and Strategies for Resource Uptake and Use

**What Factors Are Associated With Resource Uptake and Use?**	**What Strategies Could Improve Uptake and Use?**
**Sufficient training and capacity-building for users**^19^**^,^**^20^^,^^23^^,^^24^^,^^25^^–^^27^ Training and technical assistance are provided to prepare individuals to use the resource with fidelity. Training is appropriate for the audience, of sufficient duration, and integrated with existing training processes and systems. Supportive supervision is provided to all members of the team during resource use.	Offer training tailored for multidisciplinary staff. Provide ongoing training and technical assistance to support resource use. Create systems for supportive supervision. Leverage existing training systems, when possible, to introduce the resource and support its use.
**User buy-in**^19^**^,^**^20^^,^^22^^,^^23^^,^^26^ Users are aware of the resource and motivated to use it. Users believe that the resource is important, it can improve their work, and it addresses a perceived need.	Identify and leverage champions who support the resource. Articulate the rationale for using the resource, including why change is needed. Provide appropriate incentives for resource use. Consult with health care workers on resource implementation.
**Effective messaging and communication**^22^**^,^**^25^ There are effective systems and processes to support communication about resource implementation to users. The necessary systems are in place to communicate with individuals involved in implementation, including program staff. There are clear and consistent messages about the resource, its purpose, its contents, and the implementation process.	Craft clear messaging about the resource. Consider tailoring messaging to different audiences (e.g., managers and program staff). Leverage or strengthen communication systems. Establish or leverage existing communication processes with health care workers.
**Sufficient human resources**^20^**^,^**^23^^,^^25^^,^^26^ There are enough people with the time, training, and expertise to use the resource as intended.	Ensure sufficient human resources are dedicated to resource implementation. Plan for staff attrition during implementation. Evaluate the acceptability of a new resource among staff.
**Funding/financial support**^19^^,^^22^^–^^24^^,^^26^^,^^28^ There are dedicated funds and financial means, from private and/or public sources, to support resource implementation, including resource procurement, training, monitoring and evaluation, and managing unexpected delays or issues.	Evaluate whether there are sufficient dedicated funds to support sustained resource implementation. Consider multiple sources of financial support, including local and federal governments, donor, and non-health-related sectors. Provide funding to build organizational capacity before implementation. Fund critical elements of implementation, including training, supervision, monitoring and evaluation, data infrastructure, and responding to unexpected problems.
**Sufficient equipment, supplies, and infrastructure**^29^ There is enough appropriate and high-quality equipment, supplies, and other physical infrastructure to implement the resource as intended.	Ensure that there is sufficient funding to support necessary equipment and supplies to use the resource. Invest in and/or leverage national infrastructure, including transportation and information technology.
**Positive team culture**^19^ There is mutual respect across implementing team members. Team members feel part of and supported by their organization.	Prioritize understanding team culture when planning for resource implementation. Identify opportunities to strengthen or build a positive team culture. Include nonclinical staff when building a team culture.
**Leadership support**^19^**^,^**^23^ Relevant leaders of the organization, facility, and/or district are dedicated to and supportive of implementation of the resource.	Create an implementation sustainability plan with decision-makers. Establish organizational leadership’s commitment to successful implementation of the resource.
**Local and national data systems**^24^**^,^**^26^ Data are available for use and of high quality. Data are collected and reported regularly and completely. Data relevant for successful resource implementation are easily understood by resource implementers.	Provide training and capacity-building in data collection and use to resource implementers. Develop tools to support data use. Develop systems for data review and quality assurance. Leverage or strengthen national data systems.
**Political commitment and policy alignment**^22^**^,^**^29^ Government bodies and/or government personnel engage in intentional and sustained action to support resource implementation. This may include supporting or creating legislation, regulations, or rules that promote resource use. There is buy-in from national, subnational, and local health authorities for resource use. This may involve expressing support for resource use or addressing barriers.	Identify champions at various levels of government to support uptake and use of the resource. Ensure implementation of the resource aligns with laws, regulations, and policies at all levels of government. Build relationships with political officials to promote strong government buy-in for work and legislation supporting resource use. Ask governmental officials to communicate about the resource to help facilitate resource use.
**Strategic partnerships**^22^**^,^**^26^ There are relationships between 2 or more organizations or groups to share resources, information, or personnel to achieve a shared goal of supporting resource use. Strategic partnerships involving actors from the public or private sector, community representatives, and other stakeholders support use of the resource.	Identify prospective partner organizations or groups that share a common purpose related to the goals of the resource. Understand and leverage complementary skills, resources, and personnel across partnerships.

### Characteristics to Consider When Designing or Choosing a Resource

Our findings suggest that the specific characteristics of a resource may affect how well it is implemented by an individual, team, or organization. Our review found that perceived ease of use—such as familiarity with the language, layout, and overall perceived simplicity by the end user—facilitated the uptake and use of a resource.^19^^–^^22^ For example, a qualitative study on poor development and use of components of RED guidance found that end users perceived the resource as bulky and complex, which negatively affected its uptake and use.^20^ Resources should not only be in a language familiar to the end user but also include simple, concise, and clear wording to improve retention and understanding of the resource.^21^ Additionally, whether a resource is perceived as an improved, complementary alternative to the practices or tools already in use affects its implementation.^19^^,^^20^ This suggests that the value added or relative advantage of a resource compared to other alternatives is also an important characteristic. An interview respondent reflected that new resources are often duplicative of what already exists and is used. Additionally, they shared that resources developed by nongovernmental agencies are typically shared within their own networks, leading resource designers to miss out on understanding local challenges, end-user impressions and perspectives, and other critical insights that may improve the acceptability and sustainability of a resource. The evidence to support the content of the resource—or its effectiveness—was described by multiple sources as critical for resource uptake and use.^21^^,^^22^ For example, Saluja et al. found that uptake of World Health Organization guidelines for communicable diseases in LMICs was affected by the strength of recommendations and quality of evidence.^22^ Our review findings also suggest resources should be designed to be adaptable with local input so end users can modify information based on their level of knowledge, experience, needs, and environments.^20^^,^^21^^,^^23^^,^^24^ For example, a feasibility study of the World Health Organization antimicrobial stewardship resource found that the ability to adapt the resource to reflect local antimicrobial resistance surveillance data patterns and the local context supported implementation of the resource.^23^

Key informants shared that resources developed by nongovernmental agencies are typically shared within their own networks, leading resource designers to miss insights on understanding local challenges and end-user impressions and perspectives that may improve the acceptability and sustainability of a resource.

### Factors to Consider When Implementing a Resource

In addition to the characteristics of the resource itself, our findings suggest that the end user, team, organization, and larger health system contexts may impact implementation. Beyond the resource, it is critical to think about the quality of the implementation to understand how factors outside of the resource can support the efficiency and sustainability of resource use in a given context. This review found that sufficient training and capacity-building for end users to prepare for resource use was important.^19^^,^^20^^,^^23^^–^^27^ An evaluation of the implementation of RED guidance, for example, concluded that regular staff orientation, managerial capacity training, and on-the-job training were critical components of successful implementation.^25^ Additionally, Ryman and colleagues found that quality training was critical to support implementation of RED, especially in mitigating challenges such as high turnover and limited program funding.^26^ Next, we found that the end user should be motivated to use the resource, believing that it is important and that it addresses a program’s needs.^19^^,^^20^^,^^22^^,^^23^^,^^26^ Health workers in Uganda described that using microplans played a significant role in their ability to deliver routine immunization programs and accomplish program activities in appropriate timelines.^20^ Thus, user buy-in was important for resource uptake and use. We also identified effective communication, including clear and consistent messaging about resource implementation, as a factor that improves uptake and use of immunization-related resources.^22^^,^^25^ An interview respondent said that he experienced confusion regarding who was responsible for implementing or funding a routine immunization resource, which contributed to its suboptimal implementation. Across multiple sources, sufficient human capital was identified as critical to improving uptake and use of immunization-related resources.^20^^,^^23^^,^^25^^,^^26^ An evaluation of RED in Zambia found that insufficient staffing negatively affected important components of implementation of the guidance.^25^ Staff time may be needed for multiple facets of resource implementation, including monitoring and evaluation of the resource.

At the organizational level, we found that adequate funding and financial support for a facility to support the implementation of the resource improved implementation.^19^^,^^22^^–^^24^^,^^27^^,^^28^ Mala et al. reviewed multiyear national immunization plans from 77 LMICs and concluded that funding for these plans significantly influenced a country’s ability and willingness to adopt and implement them.^28^ Sufficient equipment, supplies, and infrastructure may also be important for implementation of immunization-related resources.^29^ An interview respondent shared that poor Internet connection made it difficult to communicate immunization challenges and strategies in health care facilities providing routine immunizations. The attitudes toward and support from leaders and teams implementing and using immunization-related resources are factors to consider during implementation. Our findings suggest that a positive team culture supports uptake and use of immunization-related resources.^19^ Individuals within a team should trust and respect one another when implementing and using the resource.^19^ Support from health care facility leadership was also identified as a facilitator of uptake and use of immunization-related resources.^19^^,^^23^ Evidence from key informant interviews and the literature indicated that strong facility leadership and the involvement of leadership in resource implementation improved uptake and use.^19^^,^^23^

In the broader local and national contexts, we found that accessible data systems for implementing an immunization-related resource supported uptake and use.^24^^,^^26^ For example, in Burkina Faso, poor data collection and reporting negatively affected RED implementation in routine immunization programs in 5 health districts throughout the country.^24^ Political support and policy alignment among local and national government bodies and legislation that supports the implementation of a resource were found to facilitate uptake and use.^22^^,^^29^ An evaluation of RED implementation in 70 districts in Sudan found political commitment and support for routine immunization facilitated implementation of immunization activities despite other challenges.^29^ Lastly, we found that strategic partnerships support implementation as a means to share information, resources, and personnel among organizations and key stakeholders in the private and public sectors.^22^^,^^26^ Findings suggest that creating linkages between communities, private-sector organizations, and local nonprofit organizations can help support essential components of resource implementation, such as mobilizing resources and gaining community buy-in for routine immunization programs.^26^

Political support and policy alignment among local and national government bodies and legislation that supports the implementation of a resource was found to facilitate uptake and use.

## DISCUSSION

Immunization-related resources are crucial to providing practitioners with evidence-based approaches to improve routine immunization programs and initiatives. However, the development and dissemination of such resources are not enough to ensure their use. Implementation of immunization-related resources is affected by many characteristics and factors beyond their content. The findings synthesized from the evidence presented in this article reflect early work to identify characteristics and factors needed to promote the uptake and use of immunization-related resources and can be considered a starting point for efforts to improve resource use and design to support implementation.

The findings summarized in this article may be relevant to a range of stakeholders. Moreover, different stakeholders may have the capacity and authority to act on findings differently; thus, strategies may be more or less relevant to different audiences. For example, a nation’s ministry of health may find strategies related to political commitment and policy alignment most salient, while an Essential Programme on Immunization manager may find strategies related to user buy-in and positive team culture more relevant.

Although lessons from resource implementation have been summarized in other sources, this article aims to identify and distill learnings across multiple sources to support practitioners seeking to improve resource uptake and use. A notable finding from this work is that considerations related to resource design and choice—ease of use, adaptability, and content—affect the ultimate uptake and use of the resource. This suggests that resource designers should consider how use can be supported, and decision-makers should consider design when selecting resources for use. Resource designers should ensure end users play an active and consistent role in the design, if not the origination, of the tool to ensure resources are filling a critical gap felt by end users. Arguably, emphasis should be placed on understanding user needs and perspectives and improving ease of use and relevance for the end user. Methods such as codesign, prototyping, and user testing could be beneficial in the resource design phase.

Next, adaptability to the local context emerged as an important factor for resource use, including users’ ability to ensure alignment with local policies and practices. Without guidance or clear direction, users may find it difficult to make the necessary adaptations to ensure a resource is relevant. Therefore, it may be beneficial for resources to include examples of adaptations and directions for when and how adaptations can be made. Additionally, purposeful resource design features—such as a modularized format—could facilitate more streamlined adaptation.

Without sufficient funding for implementation and sustainment, many other factors associated with uptake and use of a resource suffer. Funding is necessary for sufficient monetary incentives, training, infrastructure, and human resources. In contexts where staff time may be limited and workloads can be overstretched, it is important to consider whether program staff have the capacity to implement the resource. Similarly, sufficient well-supported human resources are necessary for a positive team culture, user buy-in, efficient training, and ongoing supportive supervision. Thus, considerations related to sustained funding are critical for promoting use. Finally, effective communication about the resource is critical to ensure user buy-in, promote a positive team culture, and secure finances to support resource use. Thus, to promote uptake and use, it may be helpful to include additional information related to communication—including key messages for different audiences—within the resource.

Effective communication about the resource is critical to ensure user buy-in, promote a positive team culture, and secure finances to support resource use.

This activity was initiated to inform planned MOMENTUM Routine Immunization Transformation and Equity project work to examine immunization-related resources to understand opportunities to improve uptake and use. The activity rapidly generated practical information that the MOMENTUM project immediately used in their immunization work. The use of findings by this team is important because it provides an opportunity to share how results can be used in practice. In their immunization work, the MOMENTUM project explored questions on resource users’ perceptions and experiences in using, implementing, and adapting a toolkit, as well as recommendations and strategies to improve the content, uptake, and use of the resource among immunization managers and other end users. To answer these questions, the MOMENTUM project created a 12-question Likert scale survey to be administered to immunization managers in several LMICs alongside an open-ended discussion guide. Additionally, the MOMENTUM project plans to use the findings to create a checklist to support resource developers in designing and revising resources. Other resource users or developers can use findings from this work to evaluate existing resources, develop new resources, or identify strategies to improve resource uptake and use within their unique contexts. For example, these findings may be important for global priorities, such as developing or implementing resources to align immunization-strengthening efforts with national primary health care called for under the World Health Organization Immunization Agenda 2030. Or they could contribute to global efforts to reverse the reductions in childhood vaccination coverage, an unintended consequence of the COVID-19 pandemic.^30^^,^^31^

Findings were generated using primarily immunization-related resources, but lessons may be applicable in other sectors within global and public health that use resources. The literature suggests a need for improved resource implementation beyond the immunization sphere. A scoping review of health-based resources that explored their use in health and health care revealed “several knowledge gaps which can inform how to best share research evidence in a way that optimizes its use.”^32^ Additionally, a qualitative study examining users of an interventional resource in a high-income country by Davis et al. found that individuals did not equate access to resources with implementation in practice—suggesting that more work is needed to ensure resource implementation.^33^ Davis et al. found that brevity and directness in a resource were associated with implementation; they also described the need for buy-in and leadership and organizational support.^33^ When assessing resource implementation and design among public health and local government officials in the United Kingdom, the adaptability and relevance of the resource to the local context, including budgets and capacity, were identified as important factors in both processes.^34^ These findings from different contexts closely align with our findings, which suggest that ease of use is an important consideration in resource design and that buy-in, leadership support, and considerations for the local context are also critical. More work is needed to understand how our findings can be broadened to new contexts in which there may also be a need for improved resource design and implementation.

Notable is the alignment between these findings and behavior change principles, which suggest that the provision of information by itself does not necessarily lead to adoption of a new behavior.^35^ Arguably, implementation of a resource is a behavior, and behavior change frameworks may provide valuable insights into other strategies for promoting resource uptake and use.

### Limitations

It should be acknowledged that this review focused on immunization-related resources used in LMICs. Our results, therefore, may not be generalizable to implementation of different types of resources or in contexts beyond LMICs. Context or resource-specific adaptations may be needed to improve relevance. Additionally, we generated our findings through a rapid review for the purposes of practice-based learning and quality improvement and did not employ research methodology, including assessing article quality. Our methods did not include a systematic literature review, which may result in selection bias. Additionally, the low response rate of practitioners invited to participate in an informational interview may result in sampling bias. Given the rapid work cycles of the MOMENTUM teams, a traditional research approach for this activity would not have aligned with the objectives and timelines of both teams. The initial findings from this work could be validated with research.

## CONCLUSION

Immunization-related resources are important tools for improving adherence to best practices that will ultimately contribute to improved immunization coverage; however, suboptimal implementation of these resources negatively impacts their effectiveness. The information in a resource is not enough to ensure that health workers and implementers use the resource. Consequently, more work is needed to identify strategies that support the uptake and use of resources. Particularly, it is critical to ensure that the intrinsic characteristics of the resource support implementation. Additionally, we must recognize factors that support resource implementation. This consideration should start at the inception of a resource; continue when a policymaker, facility, or program chooses to adopt it; and be maintained when the end user is tasked with implementation. Given the variety of contexts in which immunization-related resources are implemented, it is important to ensure such resources are adaptable to accommodate local differences. The findings outlined in this article reflect a critical first step toward providing immunization program implementers a framework for evaluating resources and adopting strategies to improve resource implementation.

## Notes

**Peer Reviewed**

**First published online:** February 9, 2024.
